# The ratio of STAT1 to STAT3 expression is a determinant of colorectal cancer growth

**DOI:** 10.18632/oncotarget.9315

**Published:** 2016-05-12

**Authors:** Harini Nivarthi, Claire Gordziel, Madeleine Themanns, Nina Kramer, Markus Eberl, Björn Rabe, Michaela Schlederer, Stefan Rose-John, Thomas Knösel, Lukas Kenner, Patricia Freund, Fritz Aberger, Xiaonan Han, Robert Kralovics, Helmut Dolznig, Susanne Jennek, Karlheinz Friedrich, Richard Moriggl

**Affiliations:** ^1^ Ludwig Boltzmann Institute for Cancer Research, Vienna, Austria; ^2^ CeMM Research Center for Molecular Medicine of the Austrian Academy of Sciences, Vienna, Austria; ^3^ Institute of Biochemistry II, University Hospital Jena, Jena, Germany; ^4^ Institute of Animal Breeding and Genetics, University of Veterinary Medicine Vienna, Medical University of Vienna, Vienna, Austria; ^5^ Institute of Medical Genetics, Medical University of Vienna, Vienna, Austria; ^6^ Department of Molecular Biology, University of Salzburg, Salzburg, Austria; ^7^ Biochemical Institute, Christian-Albrechts-University Kiel, Kiel, Germany; ^8^ Clinical Institute of Pathology, Medical University of Vienna, University of Veterinary Medicine Vienna, Vienna, Austria; ^9^ Institute of Pathology, Ludwig-Maximilians-University Munich, Munich, Germany; ^10^ Division of Gastroenterology, Hepatology and Nutrition, Cincinnati Children's Hospital Medical Center, Cincinnati, OH USA

**Keywords:** STAT1, STAT3, colorectal cancer

## Abstract

The role of STAT1 and STAT3 for colorectal carcinoma (CRC) development and progression is controversial. We evaluated 414 CRC patient samples on tissue microarrays for differential expression of STAT1 and STAT3 protein levels and correlated ratios with clinical parameters. Concomitant absence of nuclear STAT1 and STAT3 expression was associated with significantly reduced median survival by ≥33 months (p=0.003). To gain insight into underlying mechanisms, we generated four CRC cell lines with STAT3 knockdown. The cell lines harbor different known mutational drivers and were xenografted into SCID mice to analyze the influence of STAT3 on their tumor growth behavior. Experimental downregulation of STAT3 expression had differential, cell-line specific effects on STAT1 expression levels. STAT1 consistently showed nuclear localization irrespective of its tyrosine phosphorylation status. Two characteristic STAT1/3 expression patterns with opposite growth behavior could be distinguished: cell lines with a low STAT1/high STAT3 ratio showed faster tumor growth in xenografts. In contrast, xenografts of cell lines showing high STAT1 and low STAT3 levels grew slower. Importantly, these ratios reflected clinical outcome in CRC patients as well. We conclude that the ratio of STAT1 to STAT3 expression is a key determinant of CRC progression and that STAT1 counteracts pro-tumorigenic STAT3 signaling. Thus, we suggest that the STAT3/STAT1 ratios are better clinical predictors in CRC as compared to STAT3 or STAT1 levels alone.

## INTRODUCTION

Colorectal cancer (CRC) is one of the five leading causes of cancer death. CRC frequently arises from benign adenomatous polyps that can progress to carcinomas which metastasize frequently. CRC development is associated with accumulation of mutations, particularly among the components of the Wnt signaling pathway. The Adenomatous Polyposis Coli (APC) protein and nuclear β–Catenin are central components of the Wnt signaling pathway, responsible for normal intestinal epithelial cell (IEC) homeostasis. Loss of APC function resulting in elevated nuclear β–catenin is found in most CRC cases [1, 2]. Another core cancer pathway that drives CRC growth is oncogenic RAS/RAF signaling that protects cells from apoptosis [3]. The transition from benign adenoma to carcinoma is also closely associated with the loss of tumor suppressors like Smad2/3/4, p53, PTEN and changes in the microenvironment [2, 4].

Several studies have revealed aberrant JAK-STAT activity in a majority of sporadic and hereditary forms of CRC [5, 6, 7]. A subset of CRC is associated with chronic inflammatory bowel diseases such as ulcerative colitis and Crohn's disease. Patients affected with these disorders have a higher risk to develop CRC [8]. Dysregulated pro-inflammatory and oncogenic transcription factors such as nuclear factor kappa B (NF-κB) and STAT3 are common drivers of gastrointestinal cancers. NF-κB and STAT3 enhance resistance to apoptosis-based tumor surveillance of pre-neoplastic and malignant cells. Furthermore, they regulate tumor angiogenesis and invasiveness [9]. Interestingly, within the metabolic context, mitochondrial STAT3 is essential for oncogenic RAS signaling [3, 10, 11]. On the other hand, STAT1 is a prominent tumor suppressor in various cancers, but its precise role in CRC context is unclear. Interestingly, unphosphorylated STAT1 (U-STAT1) was shown to be nuclear where it is known to act on promoters of interferon-induced genes [12].

STAT3 signaling can be antagonized by STAT1 [13] in two ways: First, by forming heterodimeric STAT1/3 DNA binding complexes; second by DNA binding site competition. It was reported that STAT1 is an indicator of favorable clinical prognosis in CRC [14]. However, we have recently shown that elevated STAT3 expression is also statistically correlated with longer survival of CRC patients [15]. The mechanistic basis of these findings requires further elucidation.

In this report, we monitored the expression and activation status of both STAT1 and STAT3 in CRC patient samples and, by STAT3 knockdown in human CRC cell lines. These simulated characteristic expression/activity patterns observed in biopsies. Patients with high nuclear STAT1 and low nuclear STAT3 levels exhibited longer survival compared to patients with low nuclear STAT1 and low nuclear STAT3. We analyzed the consequences of differential STAT1 versus STAT3 expression in CRC cell lines using mouse xenograft models and observed a clear correlation with the clinical situation, i.e. STAT1/3 expression in biopsies related to overall patient survival. Two distinct response patterns upon STAT3 knockdown were observed: in two cell lines, STAT3 impairment resulted in enhanced STAT1 expression and reduced tumor growth; the other two cell lines displayed diminished STAT1 activity combined with accelerated xenograft tumor growth. Interestingly, we found that the presence of nuclear STAT1, irrespective of STAT1 activation status, is the most crucial factor influencing progression of CRC. Our study implies that assessment of total STAT1 expression, relative to the expression levels of STAT3, is an important predictive marker for the overall patient survival in CRC with diagnostic and prognostic value.

## RESULTS

### Correlation of STAT1 and STAT3 expression and activity with clinical outcome in CRC tumor tissue

**Figure 1:**
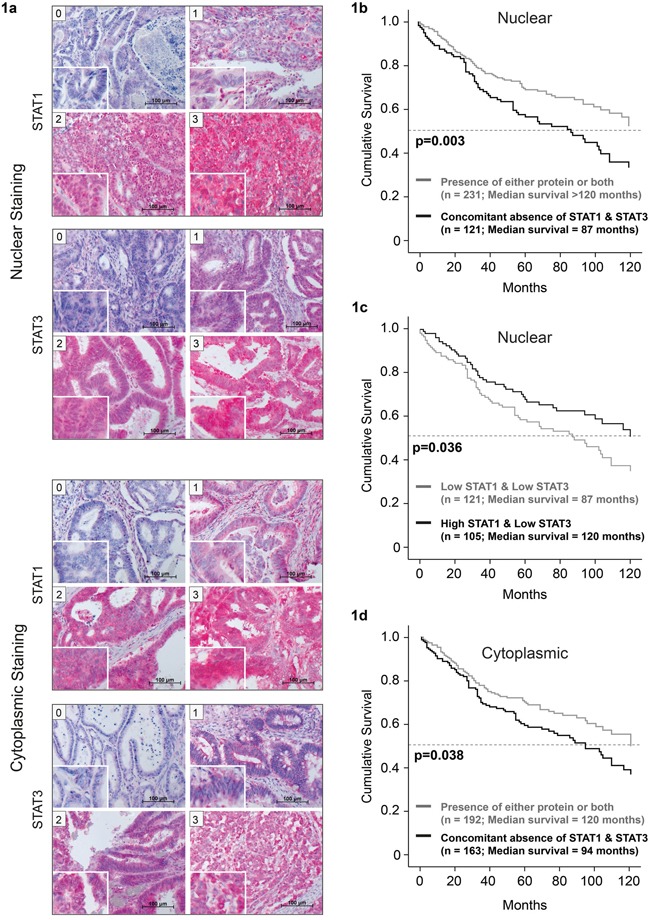
Lower median survival in patients with concomitant absence of nuclear STAT1 and STAT3 Examples of patient TMAs with nuclear STAT1 and STAT3 staining, showing different levels of STAT expression. **a.** Immunohistochemical staining was scored by two board certified pathologists as negative (score 0), weak (score 1), moderate (score 2) or strong (score 3). Patients show reduced survival upon concomitant absence of **b.** nuclear and **c.** cytoplasmic STAT1 and STAT3. **d.** Higher nuclear STAT1/STAT3 ratio correlates with increased patient survival.

We performed immunohistochemical analysis of a tissue microarray of 414 CRC biopsies from patients with full clinical documentation to check the individual and combinatorial expression and activity status of STAT1 and STAT3 in correlation with clinical information. Tissue microarrays (TMAs) were stained with antibodies against STAT1 and STAT3. The specificity of the antibodies was tested in *Stat1* and *Stat3* knockout mouse models (Supplementary methods and Figure S1). Staining for both STAT1 and STAT3 was separately determined in the cytosolic and in the nuclear compartment to distinguish between STAT expression and activity. The tissues display large differences in nuclear and cytoplasmic STAT1 and STAT3 accumulation (examples of different staining intensities; 0, 1 - ‘low’ and 2, 3 - ‘high’ are shown in Figure 1a). We found a strong correlation of combined STAT1/STAT3 parameters with overall survival of patients by separate univariate survival analysis (Figure 1b-1d). Concomitant absence of nuclear STAT1 and STAT3 as well as concomitant absence of cytosolic STAT1 and STAT3 were found to be significantly correlated with shorter overall survival of patients (p=0.003/p=0.038; Figure 1b and 1c). The median survival of patients showing neither STAT1 nor STAT3 activity (nuclear) in their tumor specimens was at least 33 months shorter in comparison to patients with tumor related activation of STAT1, STAT3 or both proteins (Figure 1b). Moreover, the median survival without STAT1 and STAT3 expression (cytosolic) was reduced by 26 months compared to patients with tumors expressing STAT1, STAT3 or both proteins (Figure 1c). Interestingly, patients with high STAT1 and low STAT3 activity had better overall survival compared to those with low STAT1 and low STAT3 activity by 33 months (increase in median survival, p= 0.036; Figure 1d).

### STAT1/STAT3 interaction and nuclear localization in CRC cell lines

**Figure 2:**
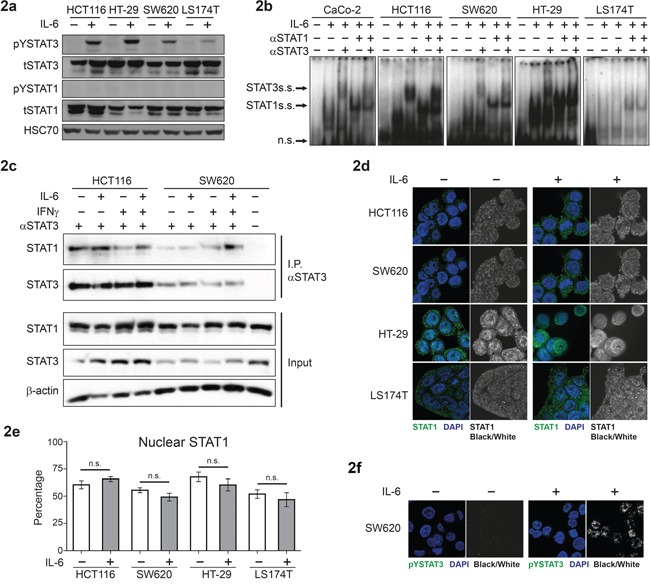
IL-6-dependent activation and subcellular localization of STAT3 and STAT1 in CRC cell lines **a.** Western blot analysis of extracts from CRC cell lines stimulated with 10 ng/ml of IL-6, for 20 min. **b.** DNA binding assay (EMSA) for STAT1/3 binding on the SIEm67 STAT1/3 response element, with CRC extracts stimulated with or without IL-6; including pre-incubation with STAT1 and/or STAT3 specific antibodies to analyse ‘supershift’ of the complexes. **c.** Immunoprecipitation of STAT3 from CRC cell lines followed by Western blot analysis with anti-STAT1 and STAT3 antibodies. **d.** Immunofluorescence and confocal microscopy for detection of intracellular localization of STAT1. **e.** Quantification of percentage of nuclear STAT1 is presented as mean values (± SEM). **f.** Immunofluorescence and confocal microscopy for detection of pYSTAT3 upon IL-6 stimulation.

IL-6 stimulation is known to lead to activation of STAT3, but at physiologic expression levels STAT1 is not tyrosine phosphorylated upon IL-6 induction in different CRC cell lines [16]. We used a panel of frequently used CRC cell lines to further investigate this observation. These five cell lines have known driver mutations, which are summarized in Supplementary Table 1). Western blot analysis showed consistent IL-6-induced activation/phosphorylation of STAT3, but no significant IL-6-dependent STAT1 tyrosine phosphorylation (Figure 2a). Notably, Electrophoretic Mobility Shift Assay (EMSA) analysis revealed the presence of STAT1 within the STAT3 DNA binding complex upon IL-6 treatment. Heterodimeric complexes containing both STAT3 and STAT1 were confirmed by DNA binding ‘supershift’ experiments in which specific antibodies to either STAT3 and/or STAT1 were added (Figure 2b).

Moreover, co-immunoprecipitation experiments, using STAT3 pulldown, revealed persistent STAT1-STAT3 interaction, irrespective of cytokine stimulation/phosphorylation status (Figure 2c and Supp. Figure S2). Next, we stimulated the four CRC cell lines with IL-6 and studied STAT1 localization by immunofluorescence and confocal microscopy. Quantification of the fluorescence intensities showed presence of 50-75% of STAT1 in the nuclear compartment. Notably, the levels of nuclear STAT1 did not increase upon stimulation with IL-6 in any of the four cell lines (Figure 2d, 2e). Importantly, the enhanced nuclear localization of pYSTAT3 upon IL-6 stimulation was clearly visible in this experimental setup, as exemplified by SW620 staining results (Figure 2f).

### Effects of STAT3 knockdown on tumor formation of CRC cell lines in xenografts

Next, we analyzed the cell lines for their ability to form tumors in SCID mouse xenografts. Apart from CaCo-2 cells, the other four CRC cell lines consistently gave rise to tumors upon sub-cutaneous injection into immunocompromised SCID mice (Supp. Figure S3).

**Figure 3:**
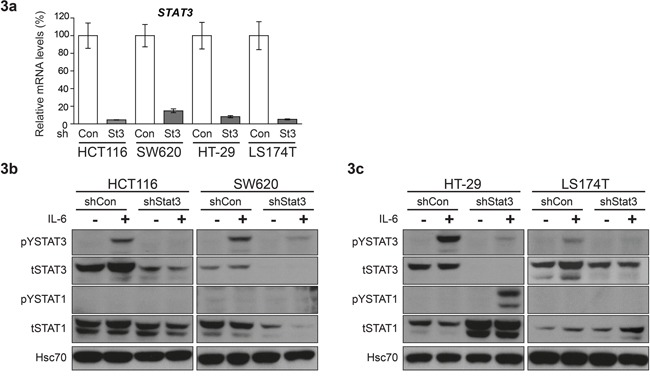
Differential effect of STAT3 knockdown on STAT1 expression in CRC cell lines **a.** Real time PCR analysis for quantification of STAT3 mRNA levels upon shRNA mediated knockdown. Mean values are shown, error bars are SEM and * p<0.05, **p<0.01, ***p<0.001. **b, c.** Western blot analysis to determine expression levels and phosphorylation status of STAT1 and STAT3 upon STAT3 knockdown in CRC cell lines.

**Figure 4:**
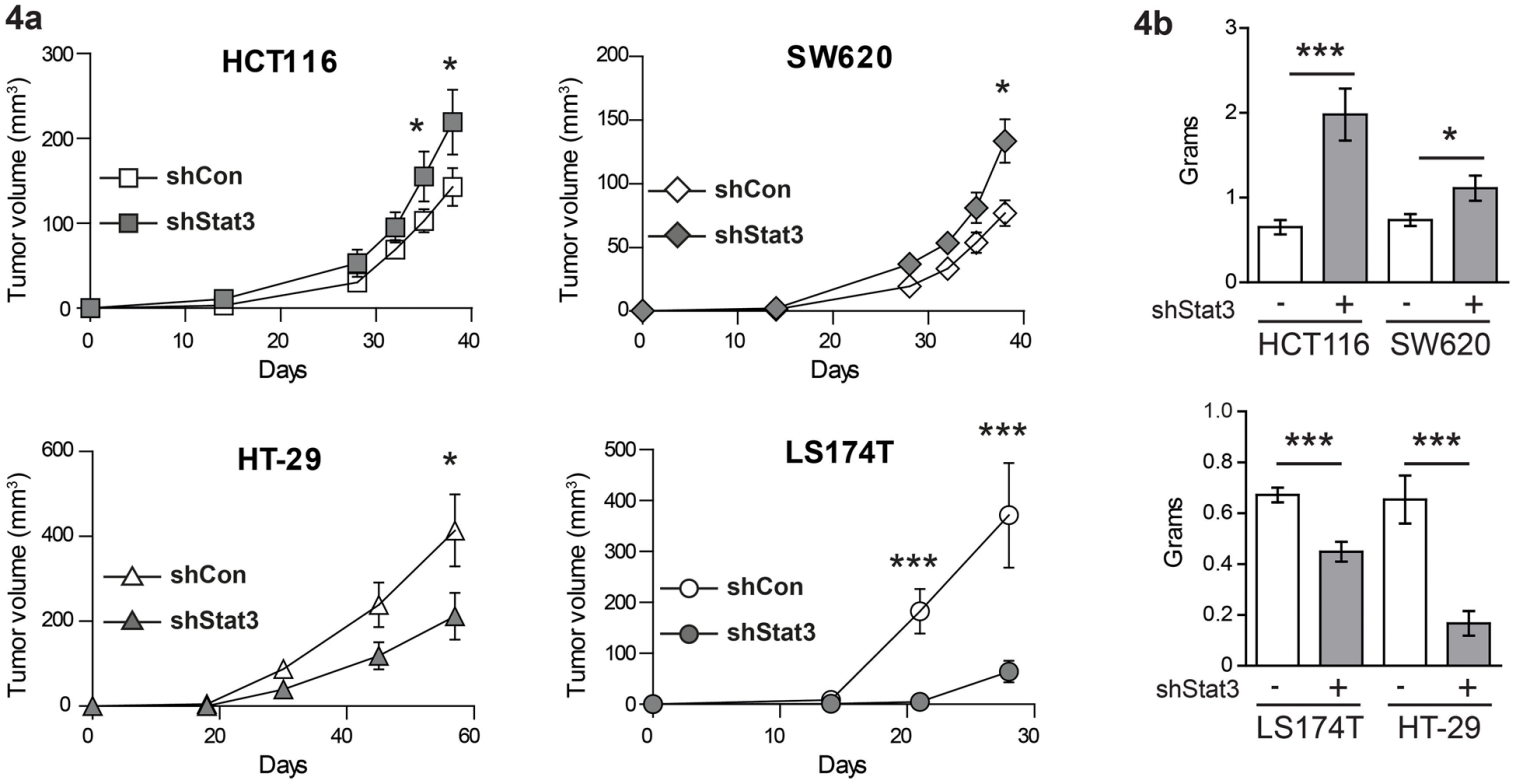
Effects of STAT3 knockdown on the growth characteristics of xenograft tumors **a.** Growth curves and **b.** end point tumor weight of xenografts of CRC cell lines with STAT3 knockdown. Mean values are shown, error bars are SEM. * p<0.05, **p<0.01, ***p<0.001.

**Figure 5:**
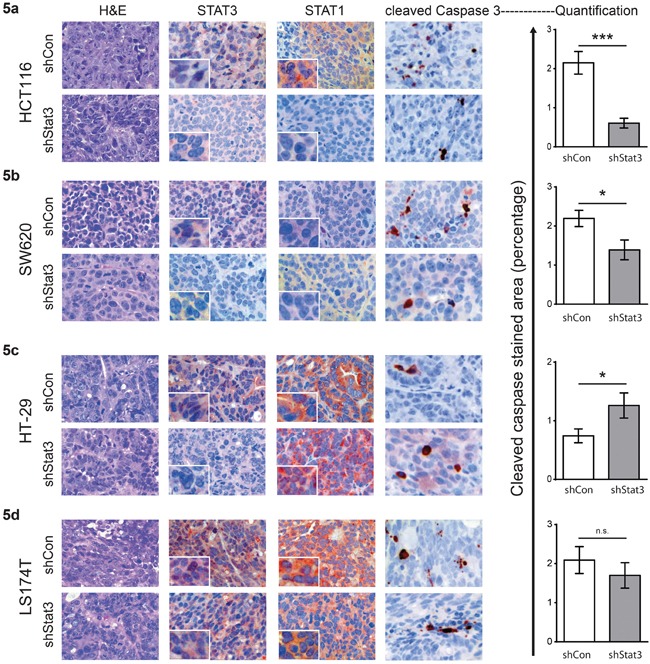
Immunohistochemical analyses of xenografted tumors of CRC cell lines with STAT3 knockdown H&E and immunohistochemical staining of xenografts for STAT3, STAT1 and cleaved CASPASE 3 for the respective cell lines – **a.** HCT116, **b.** SW620, **c.** HT29 and **d.** LS174T. Quantification of the cleaved CASPASE 3 (right panel) was done using HistoQuest TM software, from three images taken from three independent tumors. Mean values are shown, error bars are SEM and * p<0.05, **p<0.01, ***p<0.001.

In order to analyze the effects of STAT3 signaling on CRC tumor growth in xenografts, we generated stable cell lines with knockdown of STAT3, using lentiviral shRNA constructs. The reduction in *Stat3* mRNA was confirmed by real time PCR (Figure 3a) and Western blot analysis displayed significant STAT3 protein reduction in the cell lines (Figure 3b-3c and Supp. Figure S4). Interestingly, experimental reduction of STAT3 had profound effects on STAT1 expression. While STAT3 knockdown resulted in reduced STAT1 expression in HCT116 and SW620 (Figure 3b and Supp. Figure S4), it led to elevated expression levels of STAT1 in HT-29 and LS174T cells (Figure 3c and Supp. Figure S4). In line with the changes in STAT1 levels, STAT3 knockdown in HCT116 and SW620 (low STAT1+low STAT3) caused a faster xenograft tumor growth. In contrast, depletion of STAT3 in HT-29 and LS174T (high STAT1+low STAT3) resulted in growth reduction, as assessed by tumor growth curves (Figure 4a) and tumor weight at end point analysis (Figure 4b). Immunohistochemical analysis of the tumors derived from the cell lines showed patterns of STAT1 and STAT3 levels which were consistent with the Western blot and xenograft results: reduction of both STAT3 and STAT1 was observed in HCT116 and SW620 tumors (Figure 5a, 5b), while reduction of STAT3 was accompanied by an elevated STAT1 level in HT-29 (Figure 5c). Stable knockdown of STAT3 in xenografts was also monitored in tumor extracts to control for potential escape mechanism during tumor formation. Lower STAT3 expression upon shRNA STAT3 was evident in HCT116, SW620, but in HT29 and LS174T cells knockdown of STAT3 was compensated (Supp. Figure S5). This was also made evident by the reduced expression of STAT3 target genes in SW620 but not the LS174T cell line (Supp. Figure S6). Consistently, there were no discernible changes in both STAT3 and STAT1 levels in the LS174T tumors (Figure 5d). However, in the other three cell lines we detected significant changes in the levels of cleaved Caspase3 upon STAT3 knockdown in the xenografts, which correlate with the tumor growth pattern and the STAT1 expression levels in the tumors (Figure 5a-5d). In line with our results, stable STAT1 knockdown in HCT116 cells (HCT116 cells that tolerated STAT1 knockdown in contrast to our trials with LS174T, HT29 and SW620 cells where we failed to generate stable knockdown of STAT1) resulted in accelerated tumor growth in the xenografts (Supp. Figure S7). This confirmed our major conclusion, low STAT1/high STAT3 expression is associated with increased tumor growth.

### Correlation of IL-6Rα expression and patient survival

**Figure 6:**
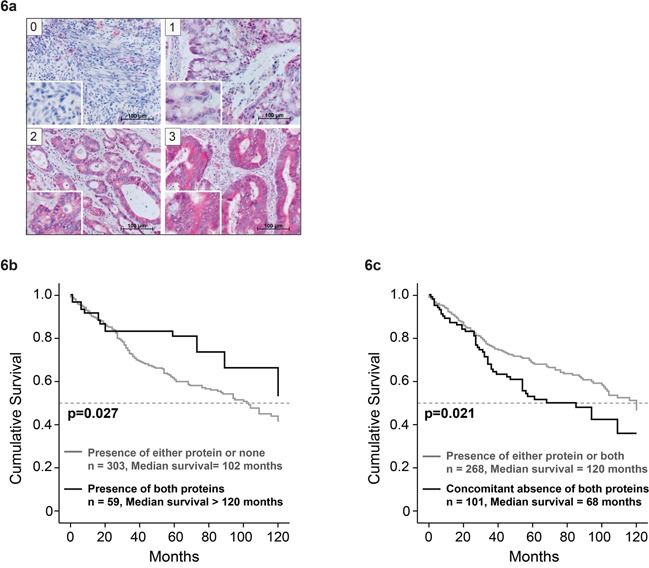
Correlation of combined STAT1 and IL-6Rα expression in CRC tissue with patient survival Examples of patient TMAs with IL-6Rα staining, showing different levels of expression. **a.** Immunohistochemical staining was scored as negative (score 0), weak (score 1), moderate (score 2) or strong (score 3). **b.** Patient survival upon concomitant presence of IL-6Rα and nuclear STAT3 and **c.** concomitant absence of IL-6Rα and cytoplasmic STAT1.

IL-6 and other members of the IL-6 family, which utilize the gp130 receptor chain as a component of their receptor complex, are involved in tumorigenic signaling. Therefore, we analyzed the CRC cell lines for expression of different receptor chains of the gp130 family. The most significant and consistent expression was observed for IL-6Rα (Supp. Figure S8). Therefore, we employed the TMAs and the associated clinical documentation to perform a combinatorial correlation analysis for the IL-6Rα, STAT3 and STAT1 expression status. Figure 6a shows the range of expression of IL6Rα in the patient tissue (scores - 0, 1 – ‘low’ and 2, 3 ‘high’). Presence of both IL-6Rα and nuclear STAT3 was found to be significantly correlated with increased patient survival (p=0.027; Figure 6b). The respective median survival was augmented by at least 18 months in comparison to patients with tumor specimens showing either only IL-6Rα or nuclear STAT3 or neither of the two. Furthermore, concomitant absence of IL-6Rα and nuclear STAT1 was significantly correlated with reduced overall survival of patients (p=0.021; Figure 6c). The median survival of patients with tumors showing neither IL-6Rα expression nor STAT1 activity was 52 months shorter than in patient groups with tumor related IL-6Rα expression and/or STAT1 activity implying that presence of Stat1 and/or IL6Rα is beneficial for patient survival.

In summary, our results are consistent with a model that suggests that the ratio of STAT1 to STAT3 expression (without the necessity of STAT1 tyrosine phosphorylation) dictates CRC tumor growth.

## DISCUSSION

We have performed a systematic study to shed light on the functional interplay of STAT3 and STAT1 and their roles in CRC growth. STAT1 is described in carcinomas as a tumor suppressor, particularly attributed to its ability to induce apoptosis and cell cycle arrest, leading to growth inhibition [17]. Indeed, tumor suppressive functions of STAT1 were associated with positive prognosis in CRC and breast cancer [14, 18, 19]. The role for STAT3 in carcinomas remains controversial - nuclear STAT3 (pYSTAT3) is regarded as a biomarker for prognosis prediction in solid tumours [20]. Several studies reported elevated and tumor promoting STAT3 signaling in CRC [5, 6, 7, 21]. In contrast, others have reported that STAT3 suppresses CRC [15, 22]. Multiple mechanisms might influence the action of STAT3 in CRC. One of these could be the presence of specific driver mutations, such as oncogenic RAS or EGFR signaling, as seen in lung cancer [23]. Another could be a change in the inflammatory status associated with specific microbiota, a specific cytokine and growth factor milieu that is also dependent on the immune cell status and the microenvironment [2, 9]. STAT1 activation may represent one of such mechanism to regulate STAT3 function. Our study was designed to clarify controversial issues regarding the role of STAT3 in CRC progression, with respect to nuclear STAT1 and STAT3 expression, interaction and hetero-dimerization. STAT1/3 have common genomic binding sites and they form hetero-dimers irrespective of tyrosine phosphorylation [24]. We show that this observation holds true also in CRC cell lines and STAT1/3 interaction exists independent of activation status (see Figure 2c). However, this interaction might be weak as it depends on low salt concentration during co-immunoprecipitation.

We did not find significant pYSTAT1 levels upon IL-6 stimulation in different CRC cell lines that were tested (Figure 2a). Only the abundance of nuclear STAT1 seems to dictate if STAT3 activity is negative for disease outcome. This finding is best exemplified by the reduced xenograft tumor growth of HT-29 and LS174T cells (Figure 4a, 4b) upon knockdown of STAT3 expression, where levels of STAT1 protein were considerably increased (Figure 3c). LS174T xenografts displayed a significant reduction in tumor growth upon STAT3 knockdown. The unaltered STAT3 and STAT1 status in LS174T xenografts at end point analysis might be due to a selective growth of cells that escaped the STAT3 knockdown, as seen in the protein and RNA expression in the xenografts (Supp. Figure S5). We observed effects on the rate of apoptosis in the xenografts of the other three cell lines corresponding to their growth rate (Figure 5). The reduction in STAT1 level upon STAT3 knockdown in HCT116 cells is mild but consistent (about 15%, Figure 3b and Supp. Figure S4). However, cells with low STAT1 expression could have a proliferative advantage resulting in significant increase in tumor growth over the time period of 40 days (Figure 4). This is also in line with the mild but significant reduction in expression of STAT1 target genes in the xenografts (Supp. Figure S9). Similarly, knockdown of STAT1 in HCT116 cells resulted in enhanced tumor growth (Suppl. Figure S7). Thus, we conclude that a higher ratio of STAT1/STAT3 expression was accompanied by better prognosis and slower xenograft growth, with smaller tumor size.

Importantly, STAT1 expression can counteract STAT3 function, even when it is not efficiently tyrosine phosphorylated (i.e unphosphorylated STAT1, U-STAT1). The U-STAT1 work was pioneered by the George Stark laboratory [12, 13] and our data suggests that the presence of U-STAT1 in the nucleus may dictate if STAT3 activity is negative for the disease outcome. It has been shown that STAT3 can transport STAT1 into the nucleus via a ‘piggyback’ mechanism and this can even overcome inactivating post-translational modifications of STAT1 [25]. It is also possible that the STAT1/STAT3 heterodimers interfere with STAT3 target gene induction via DNA binding site competition, leading to impaired CRC growth. It has been previously suggested that the mutual interdependence of STAT1 and STAT3 through heterodimer formation on the chromatin of tumor cells has a crucial influence on cancer cell fate [26, 27]. We show that STAT3 knockdown can result in variable effect on STAT1 expression levels. This variability might be explained by distinct mutational contexts (Supplementary Table 1) or other mechanisms as discussed above.

Taken together, our study provides an explanation for the controversial role of STAT3 in literature regarding CRC. Our data implies that nuclear U-STAT1 can regulate STAT3 action in a dose dependent manner. We propose that the analysis of the relative STAT1 and STAT3 expression levels is a better predictive marker for the overall survival and prognosis of CRC patients.

## MATERIALS AND METHODS

### Patients, biopsies, tumor microarray

TMAs containing samples from 414 patients were constructed. Tissue samples originated from specimens of patients who underwent surgical therapy of colorectal carcinoma in UICC stage II or III at the Surgical Department of the Jena University Hospital of the Friedrich-Schiller University. All specimens had negative margins. Data on clinical parameters, including sex, age, tumor stage, and follow-up information, were extracted from the prospective tumor registry of the surgical clinic. Pathologic findings (site of primary tumors, depth of tumor invasion, grading, lymphatic vessel invasion and venous invasion) were obtained from the pathologists' original reports. The patients were in different stages of the disease - pT1 (n=2), pT2 (n=41), pT3 (n=320), pT4 (n=51); stage I (n=1); stage II (n=204); stage III (n=209); lymph node status: pN0 (n=205), pN1 (n=130), pN2 (n=79); Grading: G1(n=203); G2 (n=159); G3 (not present). The CRC TMA was assembled using 0.6 mm punch biopsies from all 414 samples according to standard procedures as described [15, 28].

### Immunohistochemistry

Immunohistological staining of TMAs was done and evaluated as described [15]. The following commercial antibodies were used: anti-STAT1: STAT1 p84/p91 (M-22), Santa Cruz (sc-592, dilution 1:200); anti-STAT3: STAT3 (79D7) rabbit mAb, Cell Signaling (#4904, 1:400) and anti-IL-6Rα antibody (C-20), Santa Cruz (sc-661, dilution 1:200). In case of the STAT proteins, cytosolic localization was considered indicative of STAT expression whereas nuclear staining was considered a measure of STAT activation [15]. We did not obtain reliable immunostaining results with pYSTAT specific antibodies, most likely due to the phosphatases and thus, we focused on total STAT staining and localization as a reliable measure for STAT activity and expression status. All TMAs were scored twice for nuclear and cytosolic STAT staining. For statistical evaluation, scores 0 and 1 were considered as “low”, whereas scores 2 and 3 were recorded as “high”. Cleaved CASPASE 3 staining was performed as described [29]. The images were taken with a Zeiss Imager Z1 microscope and quantification was performed using HistoQuest TM software (TissueGnostics GesmbH, Vienna Austria) as described in detail [30].

### Statistical analysis of TMAs

For all data compilations, the statistics software package SPSS (version 19.0) was used. To address simultaneous activity or expression of STATs and the IL-6Rα, scores for defined pairs of protein status were combined to yield novel parameters: Co-incidence of high scores (scores 2 and 3) for both proteins was defined as “presence of both proteins”. “Concomitant absence” was defined as negative scores (scores 0 and 1) for both selected proteins. “High ratio of STAT1 and STAT3” was defined as a high score for STAT1 and a concomitant low score for STAT3. Univariate survival analysis was subsequently carried out separately for each investigated parameter applying Kaplan-Meier estimate. Survival curves were compared and assessed using the log rank test. *P* values of 0.05 or less were considered significant. All statistics were accredited by a biostatistician of the Institute of Medical Statistics, Computer Sciences and Documentation, Jena University Hospital.

### Cell culture and cytokine stimulation

The cell lines CaCo-2, HCT116, SW620, HT-29 and LS174T were cultured in DMEM (PAA) and 10% FCS (PAA). Cells were stimulated with 10 ng/ml human IL-6 (Immunotools) for 20 minutes. Extracts were made and subjected to Western blot and EMSA analysis as described [6]. Antibodies were purchased from Cell signaling (pYSTAT3 - D3A7, pYSTAT1 - #9171), BD (STAT3 - #610189, STAT1 - #610115 and Santa Cruz (HSC70 - sc-7298). For lentiviral shRNA knockdown experiments, the following Sigma-Aldrich mission TRC library constructs were used: shStat3 (TRCN0000071456) and control scrambled shRNA (SHC002). Lentivirus production and transductions were done as described previously [31].

### Immunoprecipitation

The cells were stimulated with respective cytokines (IL-6 - 10 ng/ml, IFNγ - 2.5 ng/ml) for half an hour. Protein lysates were prepared in IP buffer (25 mM HEPES, 25 mM Tris-HCl, 150 mM NaCl, 10 mM EDTA, 0.1% Tween-20, 0.5% NP-40; with protease and phosphatse inhibitors). Protein A Sepharose CL4B beads (GE Healthcare) were blocked with 2% BSA and resuspended in IP buffer. Immunoprecipitation was done overnight (at 4°C) with 1 mg of protein and 10 μg of STAT3 antibody (sc-482, Santa Cruz) in 10 mM HEPES (pH7.5) and 1 mM EDTA (5 times the volume of IP buffer). The beads were washed thrice with the HEPES-EDTA buffer. The beads were boiled with loading buffer to extract the bound immunoprecipitated protein and the supernatant was analysed by Western blotting using anti-STAT3 (BD #610189) and anti-STAT1 (BD #610115) antibodies.

### Xenografts

SCID mice were purchased from Harlan laboratories (c.b-17/icrHanHsd-Prkdc (SCID)) and received sub-cutaneous (s.c.) injections in the hind flanks with 1×10^6^ cells, resuspended in 100 μl of PBS and 0.2% BSA. The tumor size was measured with callipers and the volume was calculated by the equation 0.5 (width x width x length) [32]. The experiment was stopped before the tumor size reached 1 cm^3^ and the tumor weight was measured at the end point. All animal experiments were carried out according to an ethical animal license protocol that was approved by the Medical University of Vienna and Austrian Ministry authorities. The GraphPad Prism program was used for statistical analysis - *p<0.05, **p<0.01 and ***p<0.001 by unpaired Student's *t* test with Welch's correction. The immunohistological analysis of the tumors was done by standard procedures as described before [15].

### Immunofluorescence

Cells were washed with 1x PBS, harvested by trypsinization and 50.000 cells were seeded in each well of a 4-well chamber slide (Nunc® LabTEK® #177437). Chamber slides were incubated overnight in a humidified incubator at 37°C. The next day, the cells were treated with 10 ng/mL of rhIL6 for 20 min at 37°C and immediately fixed with 4% PFA per well for 10 min at room temperature. Cells were permeabilized with methanol for 10 min at −20°C. Slides were washed twice with 1x PBS/0.5% Tween-20 for 10 min before blocking with 1x PBS/1% BSA/0.3% Triton X-100 for one hour at room temperature. The cells were incubated overnight at 4°C with primary antibodies for STAT1 and pYSTAT3 at 1:200 in 1x PBS/1% BSA/0.5% Tween-20. After two washing steps with 1x PBS/0.5% Tween-20, cells were incubated for two hours at room temperature in the dark with secondary antibodies (Alexa Fluor 488 goat-α-mouse; goat-α-rabbit, 1:500; Invitrogen). Chambers were washed with 1x PBS/0.5% Tween-20 and incubated with 1 μg/mL of DAPI (Sigma-Aldrich) in 1x PBS for 10 min at room temperature. Slides were then washed with 1x PBS/0.5% Tween-20 and mounted using Vectashield mounting medium (Vector Laboratories, Inc.). Pictures were taken using a confocal laser scanning microscope (LSM-700; Carl Zeiss) and the ZEN 2009 software. Confocal tiff images of STAT1 immunofluorescence staining and DAPI nuclear staining were analyzed using ImageJ. The nuclear area was determined with the DAPI staining and nuclear as well as whole cell STAT1 fluorescence intensities were measured and percentage of nuclear STAT1 was calculated.

## SUPPLEMENTARY DATA FIGURES AND TABLE


