# Correction: The ratio of STAT1 to STAT3 expression is a determinant of colorectal cancer growth

**DOI:** 10.18632/oncotarget.26156

**Published:** 2018-09-18

**Authors:** Harini Nivarthi, Claire Gordziel, Madeleine Themanns, Nina Kramer, Markus Eberl, Björn Rabe, Michaela Schlederer, Stefan Rose-John, Thomas Knösel, Lukas Kenner, Patricia Freund, Fritz Aberger, Xiaonan Han, Robert Kralovics, Helmut Dolznig, Susanne Jennek, Karlheinz Friedrich, Richard Moriggl

**Affiliations:** ^1^ Ludwig Boltzmann Institute for Cancer Research, Vienna, Austria; ^2^ CeMM Research Center for Molecular Medicine of the Austrian Academy of Sciences, Vienna, Austria; ^3^ Institute of Biochemistry II, University Hospital Jena, Jena, Germany; ^4^ Institute of Animal Breeding and Genetics, University of Veterinary Medicine Vienna, Medical University of Vienna, Vienna, Austria; ^5^ Institute of Medical Genetics, Medical University of Vienna, Vienna, Austria; ^6^ Department of Molecular Biology, University of Salzburg, Salzburg, Austria; ^7^ Biochemical Institute, Christian-Albrechts-University Kiel, Kiel, Germany; ^8^ Clinical Institute of Pathology, Medical University of Vienna, University of Veterinary Medicine Vienna, Vienna, Austria; ^9^ Institute of Pathology, Ludwig-Maximilians-University Munich, Munich, Germany; ^10^ Division of Gastroenterology, Hepatology and Nutrition, Cincinnati Children’s Hospital Medical Center, Cincinnati, OH USA

**This article has been corrected:** In Figure 2C, the immunofluorescence pictures of Stat1 of the cell line SW620 were accidentally duplicated to the HCT116 cell line. The corrected Figure 2C is shown below. The authors declare that these corrections do not change the results or conclusions of this paper.

**Figure 2:**
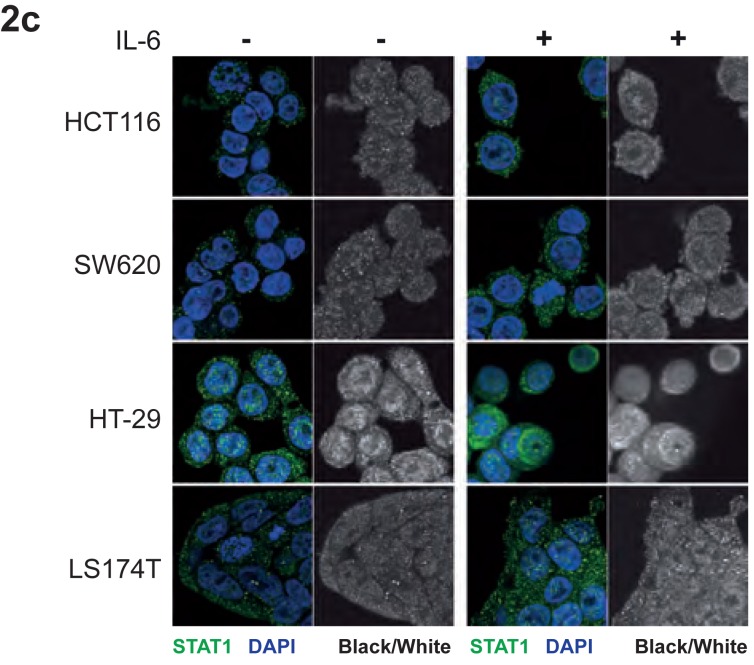
IL-6-dependent activation and subcellular localization of STAT3 and STAT1 in CRC cell lines **c.** Immunoprecipitation of STAT3 from CRC cell lines followed by Western blot analysis with anti-STAT1 and STAT3 antibodies.

Original article: Oncotarget. 2016; 7:51096-51106. https://doi.org/10.18632/oncotarget.9315

