# Supplementary material for: The ratio of STAT1 to STAT3 expression is a determinant of colorectal cancer growth
Source: Oncotarget. 2016 May 12;7(32):51096–106. doi: 10.18632/oncotarget.9315 (PMC5239461; doi:10.18632/oncotarget.9315)
Supplement: Supplementary file 1 [file oncotarget-07-51096-s001.pdf]

# The ratio of STAT1 to STAT3 expression is a determinant of colorectal cancer growth

## SUPPLEMENTARY DATA

### Staining for receptors of the GP130 family cytokines on colon carcinoma cell lines

Cells were harvested with Accutase and cell number was determined. For each antibody staining $1x10^6$ cells were resuspended in 100 µl 5% FCS/PBS. The primary antibody (see list below) was added in the indicated concentration and cells were incubated for 30 minutes at 4°C in the dark. After washing with PBS, anti-mouse IgG FITC conjugate (Sigma) was diluted 1:100 in 100 µl 5% FCS/PBS and cells were incubated with the secondary antibody for 30 minutes at 4°C in the dark. After a final wash with PBS, cells were resuspended in 500 µl 5% FCS/PBS and analysed on a FACScan.

| Antibody | Amount | Details | Positive Control |
|---|---|---|---|
| CNTFRa | 5 µl | AN-B2, Santa Cruz sc-9993 | Cos7 |
| GP130 | 10 µl | BR-3, Diaclone | 293T HEK cells |
| IL-6R | 10 µl | BR-6, Diaclone | 293T HEK cells |
| LIFR | 10 µl | Gift from Prof. Müller-Newen | STA-ET-7.2 |
| OSMRb | 5 µl | AN-A2, Santa Cruz sc-9992 | STA-ET-7.2 |

### mRNA expression levels of STAT1/STAT3 targets

1 µg of total RNA from shcontrol and shSTAT3 xenografts (n=4) was reverse-transcribed into complementary DNA using the Revert Aid cDNA synthesis kit (Fermentas, Burlington, Canada). qRT-PCR was performed using the SYBR green method. mRNA levels were normalized for beta-2 microglobulin (*B2m*), and relative abundance was calculated using the $2^{-\Delta\Delta Ct}$ method (gene-specific expression level relative to that of an endogenous housekeeping gene). Each reaction was performed in duplicate. Statistical significance is denoted as - *p<0.05, **p<0.01, ***p<0.001, Student's *t* test.

Primer sequences are listed below.

| Gene Name | Gene Symbol | Primers |
|---|---|---|
| Interferon alpha-inducible protein 27 | IFI27 | FP:GCCTCTGCTCTCACCTCATC<br>RP:CCACAACTCCTCCAATCACA |
| Bone marrow stromal cell antigen 2 | BST2 | FP:CAGAAGGGCTTTCAGGATGT<br>RP:TGATCTCTCCCTCAAGCTCC |
| Interferon-induced protein 44 | IFI44 | FP:CCTGTGCAGGGATGACATATT<br>RP:AGCGATGGGGAATCAATGTA |
| Interferon-induced protein with tetratricopeptide repeats 1 | IFIT1 | FP:CCTCCTTGGGTTCGTCTACA<br>RP:AGTGGCTGATATCTGGGTGC |
| Suppressor of cytokine signaling 3 | SOCS3 | FP:CCAAGGACGGAGACTTCGATTC<br>RP:GGAGTATTCCGGGAACCTGG |
| B-cell CLL/lymphoma 2 | BCL2 | FP:TCCGCATCAGGAAGGCTAGA<br>RP:AGGACCAGGCCTCCAAGCT |
| Myeloid cell leukemia 1 | MCL1 | FP:GGACATCAAAAACGAAGACG<br>RP:GCAGCTTTCTTGGTTTATGG |
| Hypoxia inducible factor 1 alpha subunit | HIF1A | FP:CGTTCCTTCGATCAGTTGTC<br>RP:TCAGTGGTGGCAGTGGTAGT |
| Beta-2 microglobulin | B2M | FP:GTGCTCGCGCTACTCTCTCT<br>RP:TTCAATGTCGGATGGATGAA |

## Specificity of STAT1 and STAT3 antibodies

The STAT1 antibody (Santa Cruz - sc-592) is derived against the C-terminal end of human STAT1. The STAT3 antibody (Cell Signaling #4904) is derived against the C-terminal end of mouse STAT3. Since the exact epitope is not disclosed, we performed an alignment of the last 250 amino acids of human and mouse STAT1 and STAT3 proteins using the online SIM alignment tool - http://web.expasy.org/sim/.

The following alignment parameters were used.

Comparison matrix: BLOSUM62

Number of alignments computed: 50

Gap open penalty: 12

Gap extension penalty: 4

Mouse STAT1versus human STAT1: 94.4% sequence identity

```
94.4% identity in 250 residues overlap; Score: 1240.0; Gap frequency: 0.4%

Human          1 VLSWQFSSVTKRGLNVDQLNMLGEKLLGPNASPDGLIPWTRFCKENINDKNFPFWLWIES
Mouse          2 VLSWQFSSVTKRGLNADQLSMLGEKLLGPNAGPDGLIPWTRFCKENINDKNFSFWPWIDT
                 *************** *** *********** ******************** ** **

Human         61 ILELIKKHLLPLWNDGCIMGFISKERERALLKDQQPGTFLLRFSESSREGAITFTWVERS
Mouse         62 ILELIKKHLLCLWNDGCIMGFISKERERALLKDQQPGTFLLRFSESSREGAITFTWVERS
                 ********** *************************************************

Human        121 QNGGEPDFHAVEPYTKKELSAVTFPDIIRNYKVMAAENIPENPLKYLYPNIDKDHAFGKY
Mouse        122 QNGGEPDFHAVEPYTKKELSAVTFPDIIRNYKVMAAENIPENPLKYLYPNIDKDHAFGKY
                 ************************************************************

Human        181 YSRPKEAPEPMELDGPKGTGYIKTELISVSEVHPSRLQTTDNLLPMSPEEFDEVSRIVGS
Mouse        182 YSRPKEAPEPMELDDPKRTGYIKTELISVSEVHPSRLQTTDNLLPMSPEEFDEMSRIVGP
                 ************** ** *********************************** *****

Human        241 VEFDSMMNTV
Mouse        242 -EFDSMMSTV
                  ****** **
```

Mouse STAT3 versus human STAT3: 99.6% sequence identity

```
99.6% identity in 250 residues overlap; Score: 1323.0; Gap frequency: 0.0%

Human          1 SIEQLTTLAEKLLGPGVNYSGCQITWAKFCKENMAGKGFSFWVWLDNIIDLVKKYILALW
Mouse          1 SIEQLTTLAEKLLGPGVNYSGCQITWAKFCKENMAGKGFSFWVWLDNIIDLVKKYILALW
                 ************************************************************

Human         61 NEGYIMGFISKERERAILSTKPPGTFLLRFSESSKEGGVTFTWVEKDISGKTQIQSVEPY
Mouse         61 NEGYIMGFISKERERAILSTKPPGTFLLRFSESSKEGGVTFTWVEKDISGKTQIQSVEPY
                 ************************************************************

Human        121 TKQQLNNMSFAEIIMGYKIMDATNILVSPLVYLYPDIPKEEAFGKYCRPESQEHPEADPG
Mouse        121 TKQQLNNMSFAEIIMGYKIMDATNILVSPLVYLYPDIPKEEAFGKYCRPESQEHPEADPG
                 ************************************************************

Human        181 SAAPYLKTKFICVTPTTCSNTIDLPMSPRTLDSLMQFGNNGEGAEPSAGGQFESLTFDME
Mouse        181 SAAPYLKTKFICVTPTTCSNTIDLPMSPRTLDSLMQFGNNGEGAEPSAGGQFESLTFDMD
                 ***********************************************************

Human        241 LTSECATSPM
Mouse        241 LTSECATSPM
                 **********
```

Since there is such high similarity between the human and mouse STAT1 and STAT3, we conclude that the epitope recognition in human and mouse STAT3 will be close to 100% or identical and the one in STAT1 has a high probability >94% to be also shared in these species. Both antisera are polyclonal rabbit sera and we tested the specificity of the STAT1 and STAT3 antibodies used for the TMA analysis by performing immune-histochemical analysis on mouse livers deficient either in STAT1 [1] or STAT3 [2]. Furthermore, we used positive controls for STAT1 and STAT3 staining of consecutive liver sections from mice lacking STAT5 [3] that were injected with 2 mg/kg recombinant human growth hormone (Immunotools, Friesoythe; Germany) as described [4] (Figure S1).

## REFERENCES

1. Shankaran, V., Ikeda, H., Bruce, A.T., White, J.M., Swanson, P.E., Old, L.J., et al., (2001) IFNgamma and lymphocytes prevent primary tumour development and shape tumour immunogenicity. Nature. 410: p. 1107-11.
2. Mair, M., Zollner, G., Schneller, D., Musteanu, M., Fickert, P., Gumhold, J., et al., (2010) Signal transducer and activator of transcription 3 protects from liver injury and fibrosis in a mouse model of sclerosing cholangitis. Gastroenterology. 138: p. 2499-508.
3. Cui, Y., Hosui, A., Sun, R., Shen, K., Gavrilova, O., Chen, W., et al., (2007) Loss of signal transducer and activator of transcription 5 leads to hepatosteatosis and impaired liver regeneration. Hepatology. 46: p. 504-13.
4. Schlederer, M., Mueller, K.M., Haybaeck, J., Heider, S., Huttary, N., Rosner, M., et al., (2014) Reliable quantification of protein expression and cellular localization in histological sections. PLoS One. 9: p. e100822.
5. Homfray, T.F., Cottrell, S.E., Ilyas, M., Rowan, A., Talbot, I.C., Bodmer, W.F., et al., (1998) Defects in mismatch repair occur after APC mutations in the pathogenesis of sporadic colorectal tumours. Hum Mutat. 11: p. 114-20.
6. Rowan, A.J., Lamlum, H., Ilyas, M., Wheeler, J., Straub, J., Papadopoulou, A., et al., (2000) APC mutations in sporadic colorectal tumors: A mutational “hotspot” and interdependence of the “two hits”. Proc Natl Acad Sci U S A. 97: p. 3352-7.
7. Ikediobi, O.N., Davies, H., Bignell, G., Edkins, S., Stevens, C., O’Meara, S., et al., (2006) Mutation analysis of 24 known cancer genes in the NCI-60 cell line set. Mol Cancer Ther. 5: p. 2606-12.
8. Seth, R., Crook, S., Ibrahem, S., Fadhil, W., Jackson, D., Ilyas, M., (2009) Concomitant mutations and splice variants in KRAS and BRAF demonstrate complex perturbation of the Ras/Raf signalling pathway in advanced colorectal cancer. Gut. 58: p. 1234-41.
9. Simi, L., Pratesi, N., Vignoli, M., Sestini, R., Cianchi, F., Valanzano, R., et al., (2008) High-resolution melting analysis for rapid detection of KRAS, BRAF, and PIK3CA gene mutations in colorectal cancer. Am J Clin Pathol. 130: p. 247-53.
10. Janakiraman, M., Vakiani, E., Zeng, Z., Pratilas, C.A., Taylor, B.S., Chitale, D., et al., (2010) Genomic and biological characterization of exon 4 KRAS mutations in human cancer. Cancer Res. 70: p. 5901-11.
11. Dunn, E.F., Iida, M., Myers, R.A., Campbell, D.A., Hintz, K.A., Armstrong, E.A., et al., (2011) Dasatinib sensitizes KRAS mutant colorectal tumors to cetuximab. Oncogene. 30: p. 561-74.
12. Tan, Y.H., Liu, Y., Eu, K.W., Ang, P.W., Li, W.Q., Salto-Tellez, M., et al., (2008) Detection of BRAF V600E mutation by pyrosequencing. Pathology. 40: p. 295-8.
13. Hinoue, T., Weisenberger, D.J., Pan, F., Campan, M., Kim, M., Young, J., et al., (2009) Analysis of the association between CIMP and BRAF in colorectal cancer by DNA methylation profiling. PLoS One. 4: p. e8357.
14. Jarry, A., Masson, D., Cassagnau, E., Parois, S., Laboisse, C., Denis, M.G., (2004) Real-time allele-specific amplification for sensitive detection of the BRAF mutation V600E. Mol Cell Probes. 18: p. 349-52.
15. Matos, P., Oliveira, C., Velho, S., Goncalves, V., da Costa, L.T., Moyer, M.P., et al., (2008) B-Raf(V600E) cooperates with alternative spliced Rac1b to sustain colorectal cancer cell survival. Gastroenterology. 135: p. 899-906.
16. Oliveira, C., Pinto, M., Duval, A., Brennetot, C., Domingo, E., Espin, E., et al., (2003) BRAF mutations characterize colon but not gastric cancer with mismatch repair deficiency. Oncogene. 22: p. 9192-6.
17. El-Bahrawy, M., Poulsom, R., Rowan, A.J., Tomlinson, I.T., Alison, M.R., (2004) Characterization of the E-cadherin/catenin complex in colorectal carcinoma cell lines. Int J Exp Pathol. 85: p. 65-74.
18. Ikenoue, T., Ijichi, H., Kato, N., Kanai, F., Masaki, T., Rengifo, W., et al., (2002) Analysis of the beta-catenin/T cell factor signaling pathway in 36 gastrointestinal and liver cancer cells. Jpn J Cancer Res. 93: p. 1213-20.
19. Ilyas, M., Tomlinson, I.P., Rowan, A., Pignatelli, M., Bodmer, W.F., (1997) Beta-catenin mutations in cell lines established from human colorectal cancers. Proc Natl Acad Sci U S A. 94: p. 10330-4.
20. Bryan, E.J., Jokubaitis, V.J., Chamberlain, N.L., Baxter, S.W., Dawson, E., Choong, D.Y., et al., (2002) Mutation analysis of EP300 in colon, breast and ovarian carcinomas. Int J Cancer. 102: p. 137-41.
21. Woodford-Richens, K.L., Rowan, A.J., Gorman, P., Halford, S., Bicknell, D.C., Wasan, H.S., et al., (2001) SMAD4 mutations in colorectal cancer probably occur before chromosomal instability, but after divergence of the

microsatellite instability pathway. Proc Natl Acad Sci U S A. 98: p. 9719-23.

22. Fleming, N.I., Jorissen, R.N., Mouradov, D., Christie, M., Sakthianandeswaren, A., Palmieri, M., et al., (2013) SMAD2, SMAD3 and SMAD4 mutations in colorectal cancer. Cancer Res. 73: p. 725-35.
23. MacGrogan, D., Pegram, M., Slamon, D., Bookstein, R., (1997) Comparative mutational analysis of DPC4 (Smad4) in prostatic and colorectal carcinomas. Oncogene. 15: p. 1111-4.
24. van Haaften, G., Dalgliesh, G.L., Davies, H., Chen, L., Bignell, G., Greenman, C., et al., (2009) Somatic mutations of the histone H3K27 demethylase gene UTX in human cancer. Nat Genet. 41: p. 521-3.
25. Liu, Y., Bodmer, W.F., (2006) Analysis of P53 mutations and their expression in 56 colorectal cancer cell lines. Proc Natl Acad Sci U S A. 103: p. 976-81.
26. Djelloul, S., Forgue-Lafitte, M.E., Hermelin, B., Mareel, M., Bruyneel, E., Baldi, A., et al., (1997) Enterocyte differentiation is compatible with SV40 large T expression and loss of p53 function in human colonic Caco-2 cells. Status of the pRb1 and pRb2 tumor suppressor gene products. FEBS Lett. 406: p. 234-42.

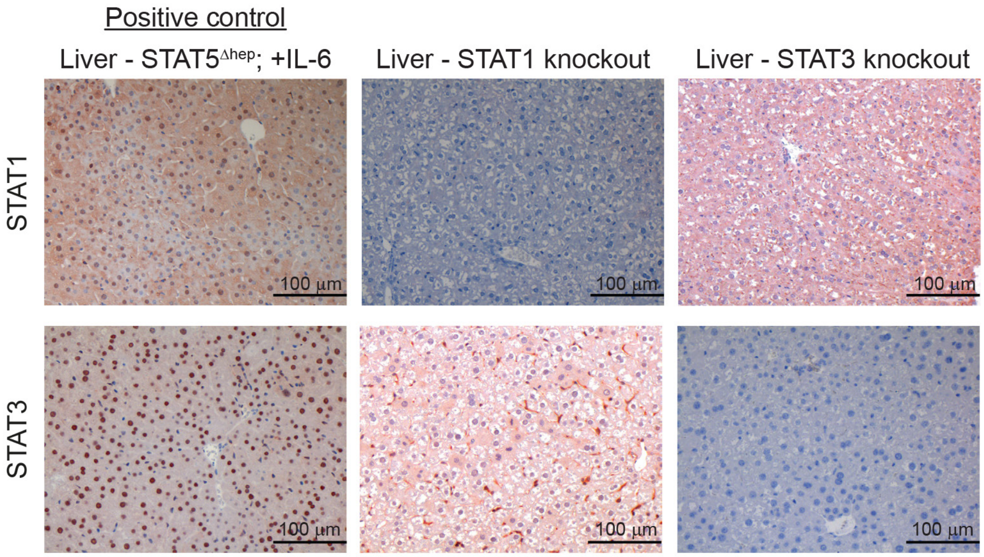


**Supplementary Figure S1: Specificity of STAT1 and STAT3 antibodies.** Paraffin embedded fixed liver samples from mice with respective genotypes were immunohistochemically stained with anti-STAT1 and STAT3 antibodies.

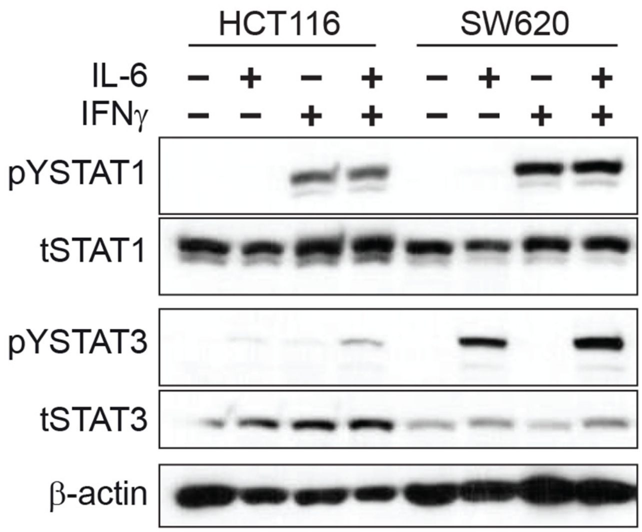


**Supplementary Figure S2: STAT1 and STAT3 activation by IL-6 and IFNγ.** Western blot analysis of HCT116 and SW620 cell lines stimulated IL-6, IFNγ and both, with anti-(pY)STAT1 and anti-(pY)STAT3 antibodies. B-actin was used as loading control.

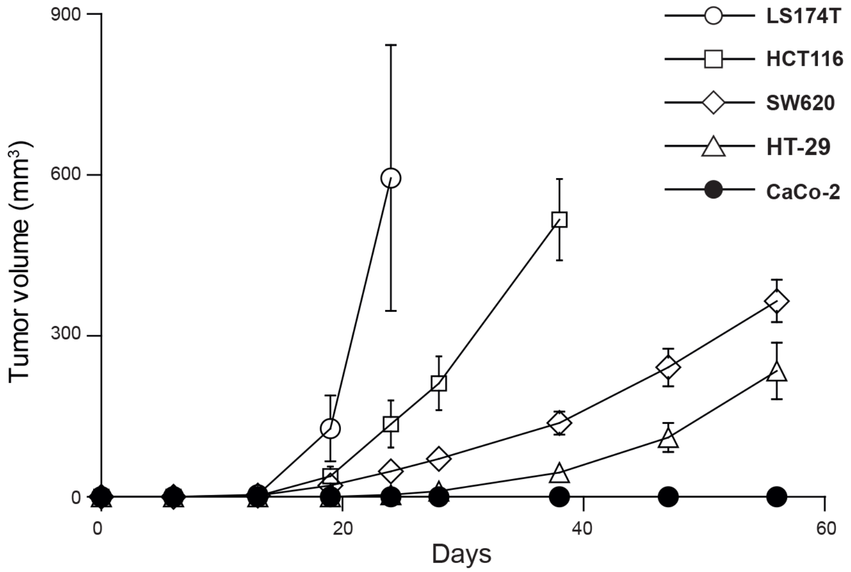


**Supplementary Figure S3: Tumor growth in xenografts by colon carcinoma cell lines.** 1 million cells of the indicated cell line were injected sub-cutaneously in the hind flanks of SCID mice and the development of tumor volume was followed for up to 60 days. Mean values are shown and error bars are SEM.

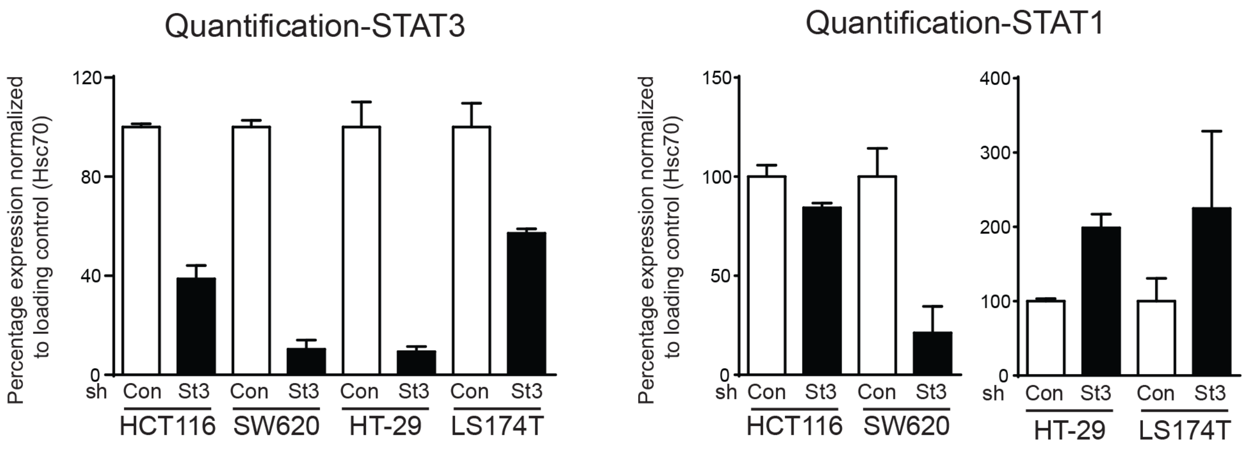


**Supplementary Figure S4: Quantification of western blots.** The open source software Image J program was used to quantify the intensity of the STAT3 and STAT1 bands in figures 3b and 3c. Mean values are shown and error bars show the range of expression levels.

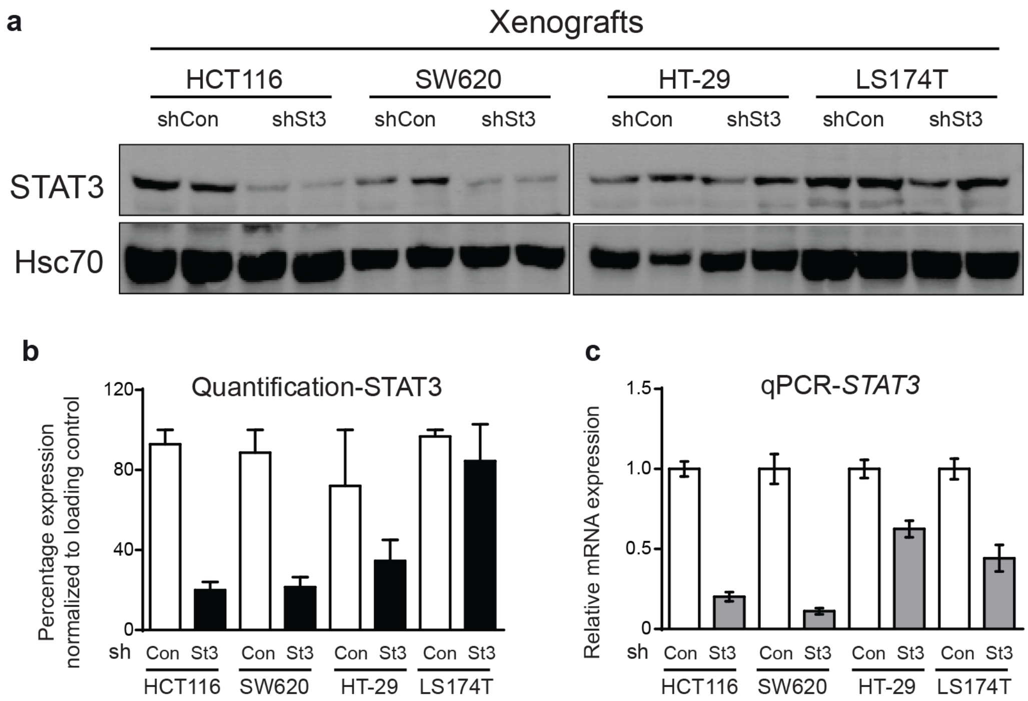


**Supplementary Figure S5: STAT3 expression levels in xenografts. a.** Protein lysates from xenograft samples derived from control and shSTAT3 cell lines were subjected to Western blot and probed with anti-STAT3 and anti-Hsc70 antibodies. **b.** Quantification of the Western blot (Image J), mean values are shown and error bars show the range of expression levels. **c.** mRNA expression of STAT3 (normalized to beta-2 microglobulin (*B2m*) mRNA) in the xenografts as quantified by RT-qPCR. Mean values are shown, error bars are SEM and * p<0.05, **p<0.01, ***p<0.001.

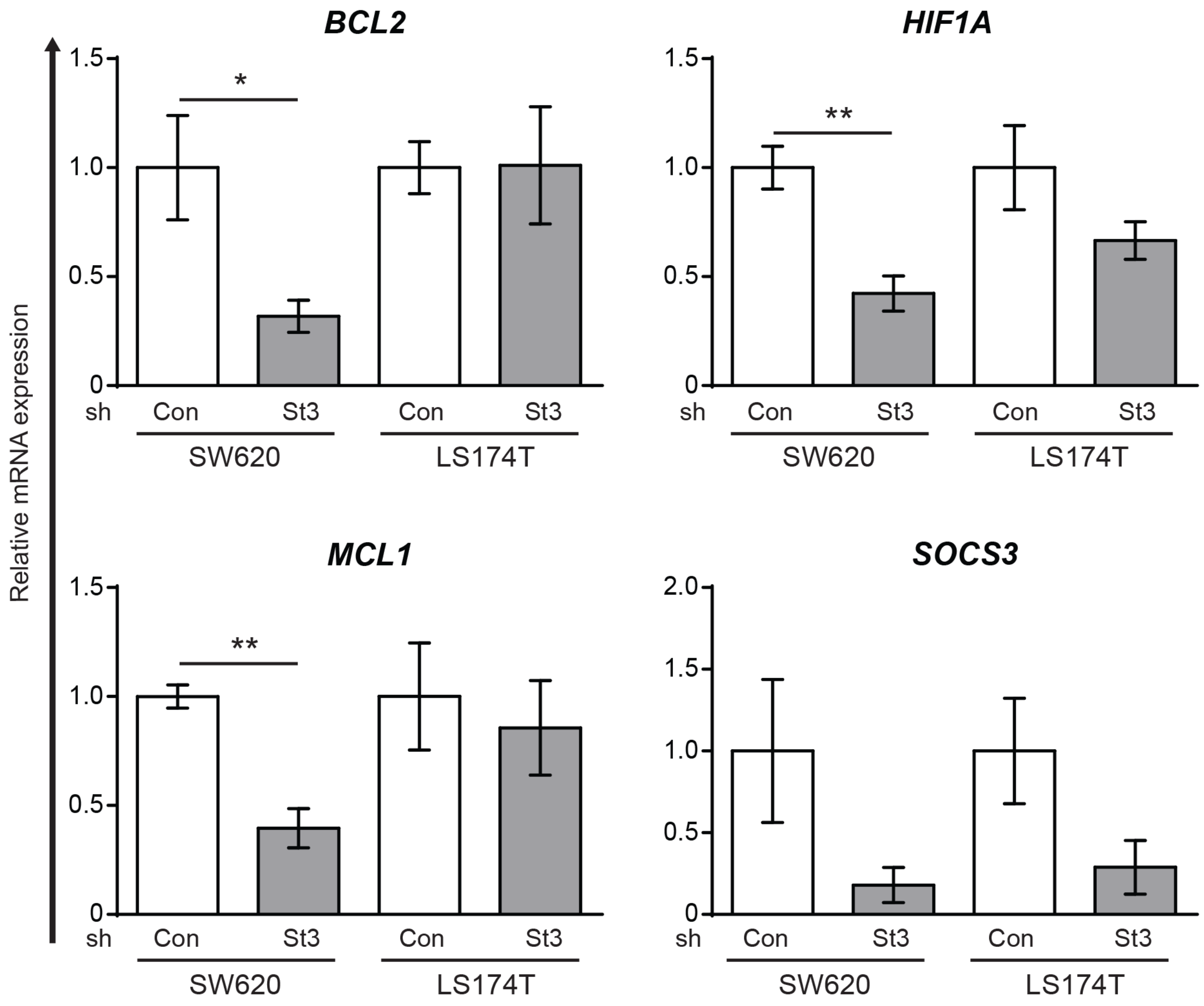


**Supplementary Figure S6: Expression of STAT1 target genes in xenografts.** Relative expression levels (mRNA) of the indicated STAT1 targets (normalized to beta-2 microglobulin (*B2m*) mRNA) was measured by RT-qPCR. Mean values are shown, error bars are SEM and * p<0.05, **p<0.01, ***p<0.001.

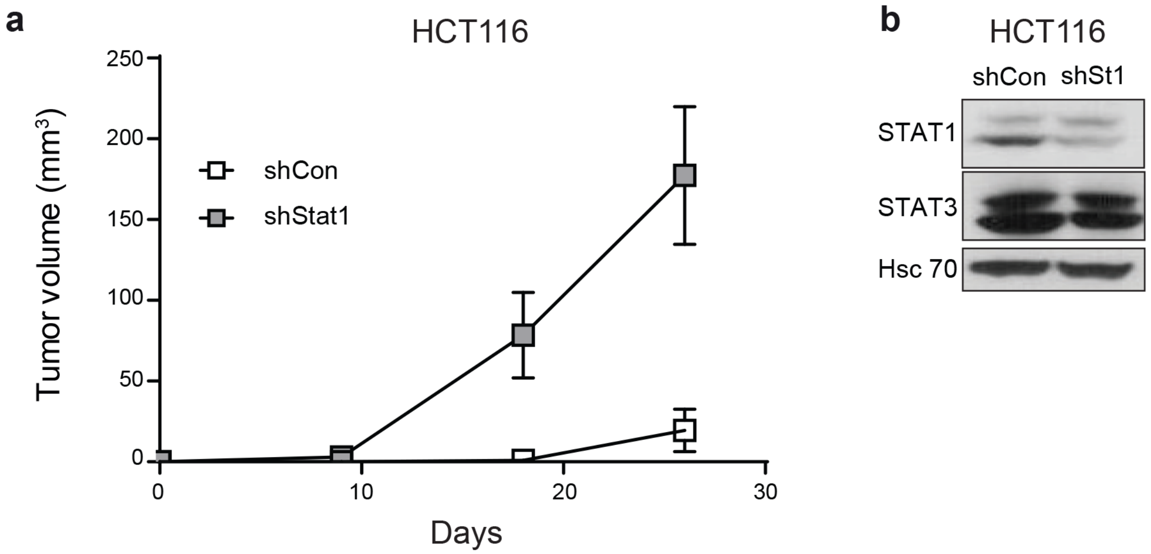


**Supplementary Figure S7: Effect of STAT1 knockdown on tumor growth of HCT116 xenografts upon STAT1 knockdown. a.** 1 million cells were injected subcutaneously in the hind flanks of SCID mice. Tumor volume was followed over time. Mean values are shown, error bars are SEM and * $p<0.05$, **$p<0.01$, ***$p<0.001$. **b.** Western blot for STAT1 and STAT3 expression in the cell lines. Hsc70 was used as loading control.

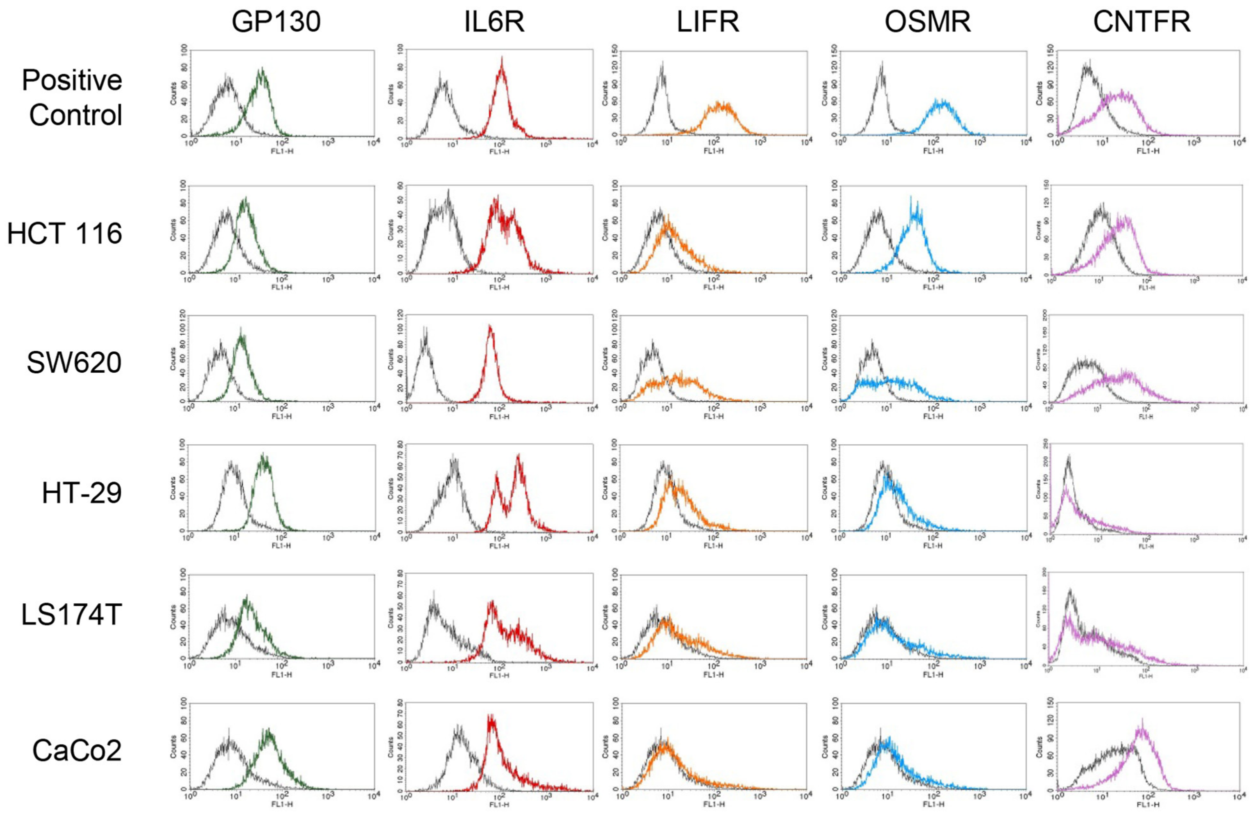


**Supplementary Figure S8: Expression of GP130 and receptors of the IL-6 family cytokines on colon carcinoma cell lines.** FACS analysis using specific antibodies to the indicated receptors with fluorescence labelled secondary antibodies (colored lines) vs. controls without primary antibodies (black lines).

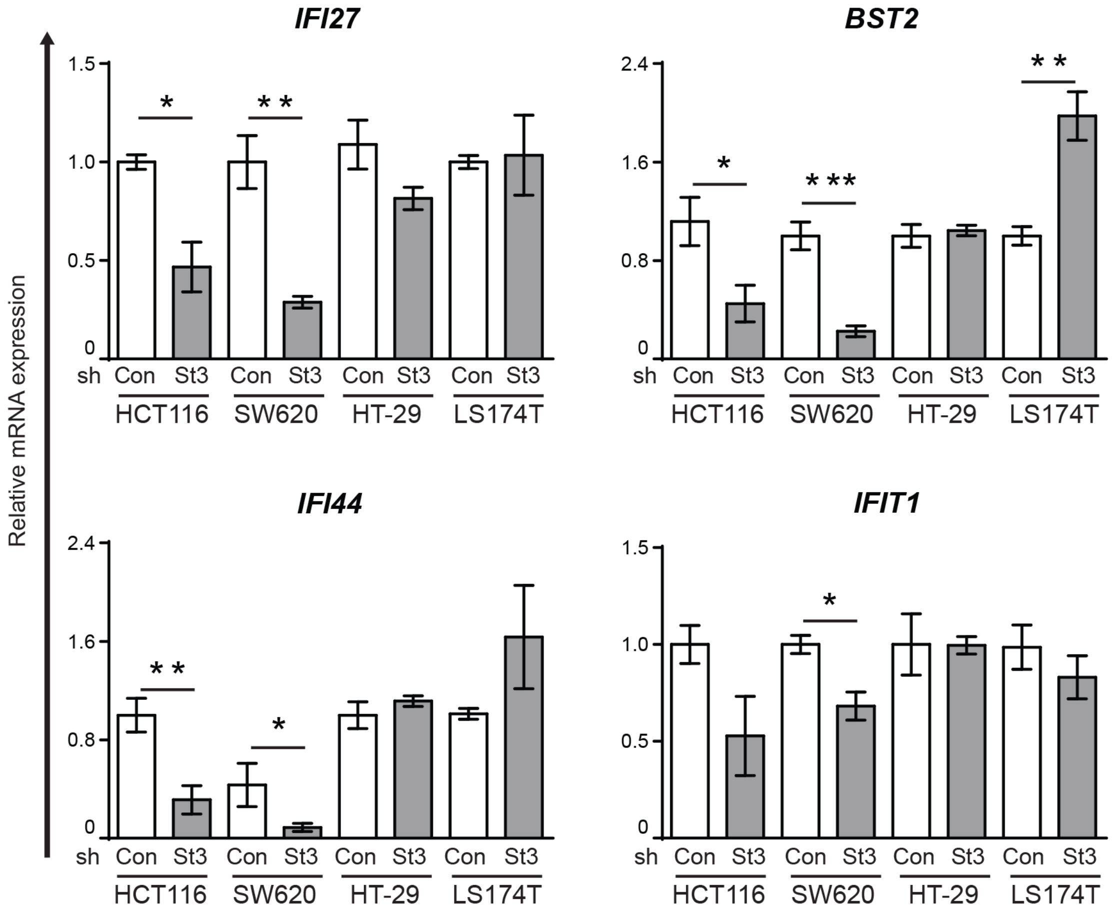


**Supplementary Figure S9: Expression of STAT1 target genes in xenografts.** Relative expression levels (mRNA) of the indicated STAT1 targets (normalized to beta-2 microglobulin (*B2m*) mRNA) was measured by RT-qPCR. Mean values are shown, error bars are SEM and * p<0.05, **p<0.01, ***p<0.001.

**Supplementary Table S1: Mutational status of relevant genes in the colon carcinoma cell lines**

| | HCT116 | SW620 | HT-29 | LS174T | CaCo2 |
|---|---|---|---|---|---|
| **Gene** | **Status** | **Status** | **Status** | **Status** | **Status** |
| APC | Wt [5, 6, 7] | p.Q1338* [5, 6, 7] | 1)p.E853*, 2)p.T1556fs* 3) p.E853* [6, 7] | Wt [5, 6, 7] | p.Q1367* [5, 6, 7] |
| KRAS | p.G12D [7] | p.G12V [8, 9] | Wt [7] | p.G12D [10] | Wt [11] |
| BRAF | Wt [7] | Wt [8, 12] | p.V600E [7] | Wt [7] | Wt [13, 14, 15, 16] |
| CTNNB1 | p.S45del [7] | Wt [17, 18] | Wt [7] | - | 1) -, 2) p.G245A [17, 18, 19] |
| EP300 | 1) p.M1470fs*3 2) p.N1700fs*9 [20] | Wt [20] | p.M1470fs*3 [20] | - | Wt [20] |
| MLH1 | p.S252* [7] | Wt [7] | Wt [7] | Wt [7] | - |
| CDKN2A (p16Ink4 p19ARF) | p.R24fs*20 p.G23fs p.E74fs*15 [7] | - | - | Wt [7] | - |
| PIK3CA | p.H1047R [7] | p.H1047R | 1) p.P449T 2) – [7] | p.H1047R [7] | - |
| SMAD4 | Wt [7] | p.? [21] | p.Q311* [7] | Wt [7] | p.D351H [21, 22, 23] |
| TP53 | Wt [7, 24, 25] | 1) p.R273H 2) p.P309S [25] | p.R273H [7, 25] | - | 1) p.Glu204X 2) – [25, 26] |
